# The eHealth Behavior Management Model: A Stage-based Approach to Behavior Change and Management

**Published:** 2004-09-15

**Authors:** Robert J. Bensley, Nelda Mercer, Judith Anderson, Deanne Kelleher, John J. Brusk, Ric Underhile, Jason Rivas, Melissa Lupella, André C. de Jager

**Affiliations:** Western Michigan University; Michigan Department of Community Health, Lansing, Mich; Michigan Department of Community Health, Lansing, Mich; Michigan Department of Community Health, Lansing, Mich; Western Michigan University, Kalamazoo, Mich; Western Michigan University, Kalamazoo, Mich; Western Michigan University, Kalamazoo, Mich; University of Illinois-Chicago, Chicago, Ill; University of Port Elizabeth, Port Elizabeth, South Africa

## Abstract

Although the Internet has become an important avenue for disseminating health information, theory-driven strategies for aiding individuals in changing or managing health behaviors are lacking. The eHealth Behavior Management Model combines the Transtheoretical Model, the behavioral intent aspect of the Theory of Planned Behavior, and persuasive communication to assist individuals in negotiating the Web toward stage-specific information. It is here — at the point of stage-specific information — that behavioral intent in moving toward more active stages of change occurs.

The eHealth Behavior Management Model is applied in three demonstration projects that focus on behavior management issues: parent-child nutrition education among participants in the U.S. Department of Agriculture Special Supplemental Nutrition Program for Women, Infants and Children; asthma management among university staff and students; and human immunodeficiency virus prevention among South African women. Preliminary results have found the eHealth Behavior Management Model to be promising as a model for Internet-based behavior change programming. Further application and evaluation among other behavior and disease management issues are needed.

## Introduction

It has been 30 years since the release of *A New Perspective on the Health of Canadians: A Working Document* (1). This report solidified the role of lifestyle behaviors as the most likely cause of premature death and sparked a flurry of international efforts toward preventing and reducing the occurrence of behaviors known to be determinants of morbidity and mortality. For the past 30 years, health-risk behavior has continued to be a prime focus of public health efforts. The leading causes of death in the United States are dominated by health-risk behaviors — with tobacco use, poor diet, physical inactivity, and alcohol consumption contributing to nearly 40% of all U.S. deaths (2).

As a greater focus on behavioral determinants of health evolved, so did the development and implementation of theories and models of behavior. Focus has increased on individual health behavior theories and models such as the Theory of Planned Behavior, the Health Belief Model, and the Transtheoretical Model. The Transtheoretical Model is one of the most popular models for describing the processes that individuals undergo as they move through stages of readiness to change behaviors (3).

It is not the intent of this paper to provide an in-depth discussion of the merits of behavior change and prevention of premature death. It is also not the intent to compare and contrast the myriad behavior change theories and models. Rather, the purpose of this paper is to present the eHealth Behavior Management Model, a behaviorally focused model that applies existing theories to one of the newest and greatest potential avenues of intervention: the Internet (4).

In a little more than a decade, the Internet has grown from a collection of remote pages to an unparalleled information source. By 2003, it was estimated that more than 600 million users online worldwide were accessing more than 170 million Web sites (5,6). The number of individual Web pages is difficult to determine. At least three billion pages currently exist, based on the size of the Web page index for Google, the largest search engine in the world (5). The number of health-related Web pages in a simple Google search using the keyword "health" netted nearly 250 million Web pages.

The Internet is used by a variety of populations. For example, more than 20% of people over the age of 65 have reported using the Internet, with two out of three having used the Internet to seek health information (7). Populations typically perceived to be hard to reach, such as lower-income populations, are also using the Internet. One of the projects described in this paper found that nearly 60% of the 20,000 lower-income participants who accessed an Internet-based behavior change project did so from their own homes (8). This high rate of home-based computer access among lower-income populations has also been observed elsewhere (9). The U.S. Department of Health and Human Services recognizes the growing importance of the Internet as a means for accessing health-related information. Baseline data for *Healthy People 2010* show that an estimated 26% of U.S. homes had access to the Internet in 2000; the goal of *Healthy People 2010 *is to increase this percentage to 80% by the year 2010 (10). Recent polls by Internet watchdog groups have found that nearly two out of three American adults have Internet access, with more than 85% of those connected from home (11-13).

The Internet has fast established itself as a leading source for health information. It is still in its infancy, however, in how health information is accessed and used for behavior change purposes. Today, most Web-based health information exists in a resource-list format. A health-related Web page commonly consists of information with hyperlinks to other similar or complementary information. The use of search engines and portals categorizing information by topic provides users with easy ways to gain access to health information. In addition, numerous health-related behavior change approaches exist on the Internet, from basic health-risk assessment to individual behavior change approaches for challenges such as smoking cessation, albeit at basic levels of generic and broad health messages (14,15). What is missing, however, is the infusion of theoretical models and approaches, especially those oriented toward behavior change, for creating interactive and didactic communication systems that access existing Internet-based health information. Efforts to use technology for impacting health behavior have been most prolific in tailored health communication.

Tailoring is a process for creating individualized communication. This paper does not delve into the merits or shortcomings of tailored health communication, but rather explains how the eHealth Behavior Management Model fits within the broader ehealth behavior change movement. In tailored health communication, user-provided personal data are used to determine the most appropriate information or strategies for meeting the unique needs, wants, and interests of each user. Technology has allowed tailored health communication to proliferate, expanding the potential of this approach to meet the unique needs of individuals (16,17). Researchers have applied tailoring techniques to numerous behavioral issues, including childhood immunization, weight loss, smoking cessation, nutritional consumption, and sedentary behavior (18-22).

Tailored health communication relies on the ability to match individual health needs with a set of predeveloped messages. Data management and access become central issues in tailored health communication because of the ability to address the unique needs of each individual within the system. Managing large populations and their needs requires software programs capable of sorting through and matching appropriate messages with unique needs. The Internet has extended the capability of tailored communication by providing expanded and easy access to interactive assessment and delivery of messages (16,17).

The main difference between tailored health communication approaches and the eHealth Behavior Management Model is that while the former creates messages for individuals based on assessed need or interest, the latter matches behavior change messages with information that already exists on the Internet. The eHealth Behavior Management Model assesses individual need based on readiness-to-change behavior and matches the individual to preselected Web sites. Although the eHealth Behavior Management Model is still in the early stages of application, initial findings suggest that it is a promising model for Internet-based behavior change programming.

## Overview of the eHealth Behavior Management Model

The eHealth Behavior Management Model uses the theoretical underpinnings of two popular behavior change models, the Transtheoretical Model and the behavioral intent aspect of the Theory of Planned Behavior, as well as the concepts of persuasive communication, to engage Internet users in a pseudocounseling approach. A brief discussion of the key ideas behind these theories will help in understanding the eHealth Behavior Management Model*.*

The Transtheoretical Model is well known among behavioral scientists and has been widely applied in efforts designed to reduce the occurrence of a multitude of behaviors. This model provides practitioners with an effective way to move individuals toward healthier lifestyles. Change is viewed as a process that occurs over time, rather than an action that must occur as an immediate and direct result of an intervention. The Transtheoretical Model is based on the premise that individuals are in one of five possible stages of change associated with a particular behavior. Precontemplation is the stage in which a person has no interest in changing the behavior. Contemplation is when a person would like to change the behavior someday but is not yet ready. Preparation is when a person is ready to make the change but needs assistance in moving that want into reality. The more active stages include Action and Maintenance. Those in Action have begun the behavior change process. Key to their success is moving the change to Maintenance, where change takes place over time (23).

The behavioral intent aspect of the Theory of Planned Behavior is based on the premise that individuals with a defined intent toward change are more likely to engage in change, especially individuals with an attitude that change is necessary. The premise also includes the idea that the closer the change relates to subjective norms, the greater the likelihood that a sense of behavioral control exists (24). Central to this theory is that beliefs about the behavior, control, and norms influence attitude, subjective norms, and behavioral control. The theory suggests that creating a situation in which intent is high will result in a greater likelihood that an individual will carry through with the intent.

The persuasive communication concepts of response dependence and transactional interaction are key components of the eHealth Behavior Management Model. Transactional communication involves the flow of information between a persuader and a persuadee (25). It is transactional because at least two parties are involved in both sending and receiving information. To be persuasive, communication must 1) motivate receivers to attend to messages, 2) solicit feedback from users, and 3) elaborate message arguments to move individuals toward change. Current Internet resources typically utilize a one-way communication mode: information exists on a Web page and is accessed by a user. Feedback loops — in which each party involved shares in the roles of sender and receiver  — do not usually exist in Internet health communication.

Response-dependent communication controls the flow of the communication process, where responses to inquiries serve as the basis for driving the next round of persuasive messages and inquiries (25). Response-dependent communication is similar to what may occur between a counselor and a client: questions are asked based on responses to previously asked questions. The response then becomes critical to the direction in which the communication unfolds and can take many forms, such as a question or a persuasive statement, rather than simply an answer to a question.

**Figure 1:**
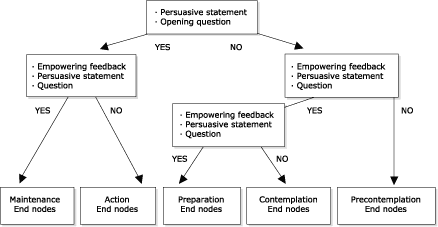
Algorithm for eHealth Behavior Management Model.

The eHealth Behavior Management Model creates a persuasive communication environment in which a user and a computer serve as two parties in the communication stream. An underlying algorithm controls the flow of communication that results from transactions between the two parties (Figure 1). The staging algorithm developed for the eHealth Behavior Management Model was developed specifically for this model and is based on approaches previously used in numerous interventions using the Transtheoretical Model, but it is yet to undergo a comprehensive validation study. The initial Web page in the algorithm consists of a persuasive statement about the issue followed by an opening question for the user. The user has the option of selecting a response, which is typically "yes," "no," "not really," or "sometimes," by clicking on the appropriate response. Subsequent Web pages in the algorithm contain empowering feedback followed by an additional statement about the issue (persuasive argument) and a subsequent question. The communication that occurs between the user and the computer is transactional in that give and take exists between both parties, and the communication is response-dependent as well in that algorithm paths are followed according to responses and feedback.

The transactional, response-dependent communication continues until the user arrives at a specific stage of readiness to change. The breadth of the popularity of the Transtheoretical Model has culminated in numerous uses of staging algorithms for myriad behaviors. A popular approach is to use a serious of yes/no questions to determine current stage and the temporal distance of the behavior, resulting in identification of a specific stage of readiness to change (26-29). The questions used in the communication process direct the user toward a specific stage of change (Figure 1). In essence, the model is acting as a diagnostic tool in determining user stage of readiness to change. At this point, the model directs the user to stage-based information already existing on the Internet. These "end nodes" contain information on the behavioral issue. Ideally, end nodes already exist on the Web. All end nodes must be reviewed and approved by model designers for credibility and appropriateness for stage of change and target population.

End-node assessment for stage of readiness to change as well as processes of change that users typically undertake to move toward more active stages are essential to this model because at this point — within the end node — critical information on the user's need for change exists. A systematic four-step method is used by model designers to identify a pool of potential end nodes: 1) review and selection, based on known association and credibility, of Web sites linked from resources on the behavior issue, such as the American Dietetic Association, the U.S. Department of Agriculture, or university extension services; 2) review of Web pages used in existing literature and other academic sources; 3) solicitation of related end nodes from a panel of expert reviewers; and 4) comprehensive search of the Internet using the leading Internet search engines and techniques.

Potential end nodes are scrutinized using a five-step process once the pool has been established. First, end nodes are reviewed to determine their direct associations with the behavioral issues. Suspect end nodes, including those that violate standards set by the U.S. Department of Health and Human Services Science Panel on Interaction Communication in Health, are eliminated. Second, end nodes are assessed for stage of readiness to change, based on general criteria of the Transtheoretical Model. End nodes that cross numerous stages are eliminated from the pool, unless a mechanism exists within the Web page to direct users to information specific to the stage. Third, potential end nodes are further matched against the specific concepts associated with the behavioral issue. Fourth, potential end nodes are assessed for the 10 processes of change associated with the Transtheoretical Model, ensuring the process of change within the end node is appropriate for both the concept associated with the behavior and the stage of change itself. Finally, end nodes are subjected to an expert panel for review and ranking, if more than one end node exists for a specific behavioral concept. End nodes that meet all five criteria are considered viable options for the eHealth Behavior Management Model.

**Figure 2:**
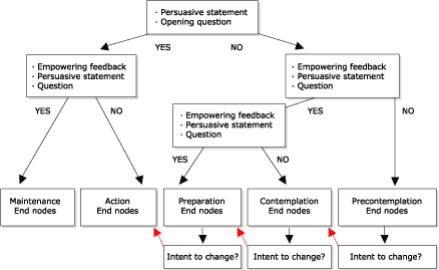
Intent to change option of the eHealth Behavior Management Model.

Also central to the model is the ability to move forward in intent toward change based on the stage-based information contained in the end node. The model allows for recognition of this intent and will adjust users in the communication stream to the next stage of readiness to change, providing users with the opportunity to fortify intent even further (Figure 2). Users also have the option of exiting from the model after reviewing stage-based end nodes. A feedback loop in the model allows users to provide comments on what continues to hold them back from moving forward in intent toward change; this feedback can provide model designers with direction on end-node selection. In the nutrition education program discussed in this paper, information on the beginning node, the ending node, and the reasons for not progressing further in stage of change are entered onto a certificate of completion, which is then used by agency staff to further counsel clients toward additional resources designed to aid in progress toward an active stage of change.

## Application of the eHealth Behavior Management Model

The utility of the eHealth Behavior Management Model is promising and may be applicable to a number of behavior management issues, including behavior change, chronic disease management, and disease prevention. Three demonstration projects will be described to show how the model is currently being applied to behavior management issues. The intent of this paper is to provide examples of how the eHealth Behavior Management Model has been adapted to fit behavior management and behavior change needs; evaluative findings of the projects described below will be presented in the future.

### Women, Infants, and Children (WIC) nutrition education

Nutrition education and nutrition counseling are vital components of the U.S. Department of Agriculture WIC Program (Special Supplemental Nutrition Program for Women, Infants, and Children), accounting for 17% of state WIC operating expenses. All WIC clients must be offered nutrition education, much of which centers on parent-child feeding behaviors, as part of their WIC participation. Traditional education methods, such as one-on-one counseling, group education, classes, and printed materials are typically used to provide this education. Educational approaches are usually topic- or skill-based and available to whomever may attend the WIC clinic on any particular day. It is not feasible or routinely practiced to segment audiences according to skills needed or stage of readiness to change. Educational offerings may be divided by age or type of participants (e.g., infants, toddlers, pregnant women, breastfeeding women) but typically adhere to a one-size-fits-all educational approach given at the Action stage of change. Many WIC clients are unable to meet for traditional nutrition education classes because of work or school schedules or transportation issues. In addition, they may not attend classes because they may find that classes do not meet their interests or needs, the educational materials offered are not appropriate for them, or their available time is used in waiting for coupons to be processed; if they do attend classes, they may be distracted by restless children. An incentive for completing secondary education is the link between WIC contact and dispersion of food coupons. While WIC benefits may not be withheld if a participant refuses or cannot attend nutrition education, clinics usually schedule nutrition education at the same time as coupon pickup to encourage attendance.

The need for more targeted stage-based educational approaches led to the development of www.wichealth.org*, an Internet-based parent-child feeding-behavior project based on the eHealth Behavior Management Model*.* The project is in its second year of implementation. To date, it has provided stage-based secondary nutrition education to more than 30,000 WIC participants in seven states. Five modules focus on providing regular meals and snacks, working with picky eaters, providing a positive feeding environment, and outlining the roles of parents and children in the feeding relationship. Nearly 70% of user access occurs from home or work, with an additional 16% occurring from parents' or friends' homes (30). First- and second-year evaluations have consistently demonstrated high levels of user satisfaction with the helpfulness and ease of use of the site (84–99%), belief in ability to make changes with what has been learned (87–90%), and interest in using the Internet to learn about other WIC topics (84–86%). Results also find the site to be popular with participants in the more active stages of readiness to change (Maintenance, Action, and Preparation), with significant movement in intent toward Action in all modules where clients started in earlier stages. Qualitative data support the quantitative findings of this project (8,31).

**Figure 3:**
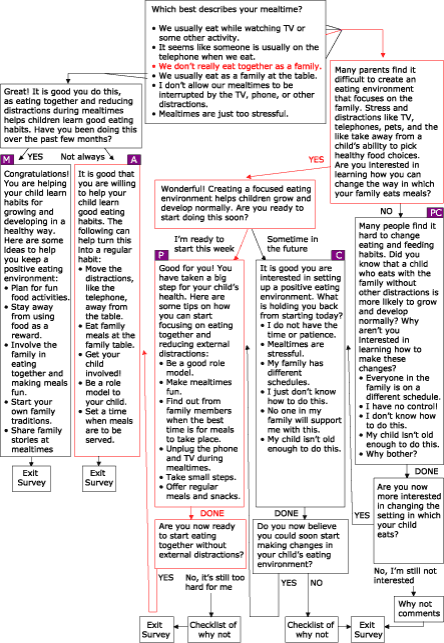
Feeding-environment skills algorithm. PC = Precontemplation; C = Contemplation; P = Preparation; A = Action; and M = Maintenance.

All three theoretical underpinnings of the eHealth Behavior Management Model are inherent in www.wichealth.org*. The module "Create good eating habits in your child" will be used as an example to depict how the eHealth model is applied to this project. Once a user selects this module for learning, the staging process, which uses persuasive communication techniques, begins with a simple question. Users in more active stages (Action-Maintenance path) are separated from those in earlier stages (Preparation-Contemplation-Precontemplation path). Both options are response-dependent and include a persuasive empowering statement and subsequent staging question, further defining the starting stage of the user. Users who initially select less active stage options such as "We don't really eat together as a family" are further separated by stage with a subsequent staging page. A positive response links to a page (Preparation-Contemplation path) that uses a transactional and response-dependent process to separate further Preparation and Contemplation stages. A subsequent response such as "I am ready to start this week" identifies the user as beginning in the Preparation stage. The system then provides a list of topics and end-node links pertinent to processes of change for WIC populations struggling with moving from Preparation to Action in child-feeding environment issues. At this point, stage-based learning takes place. Users who complete end-node review are then given the option of continuing to progress in stage of change, based on behavioral intent, or exiting the system. Those who intend to move forward enter the Action stage and have the option of learning action-oriented skills from end nodes associated with this stage of readiness to change. Those who choose to exit the system are provided with a certificate of completion as evidence of successful completion of the module. Figure 3 depicts the full staging algorithm, complete with response feedback (e.g., "Wonderful!"), empowering persuasive argument statements (e.g., "Creating a focused eating environment helps children grow and develop normally"), and subsequent staging question (e.g., "Are you ready to start doing this soon?"), with the paths used in this example depicted in red. A review of the Web site provides further perspective on how technical capabilities, such as frames and links, stage users and move them toward active stages of readiness to change.

### Chronic disease management: Asthma Management Demonstration Project

According to the Centers for Disease Control and Prevention (CDC), more than 20 million people in the United States have been diagnosed with asthma, with 12 million of them experiencing uncontrolled asthma (32). People diagnosed with asthma often accept a lower quality of life. This lower quality of life includes missing days of work or school, little or no participation in physical activity including sports, regularly interrupted sleep, and overuse of quick-relief medications.

Despite an increase in data that elucidates the prevalence, severity, and financial costs of uncontrolled or poorly managed asthma, only a small amount of data describes the unique challenges to asthma management in college or university settings. Colleges and university settings require students and employees to encounter myriad triggers of asthma. Those triggers include having little control over living environments, including residence halls and college apartments; sharing limited living space with one or more roommates; changing environments throughout the day because of classroom scheduling; and managing exposure to outdoor pollens and molds created by the vast grounds-keeping activities necessary for college campuses (e.g., mowing), to the prevalence of tobacco smoke on college campuses, and to fumes emanating from chemistry labs, art studios, and physical plants.

**Figure 4:**
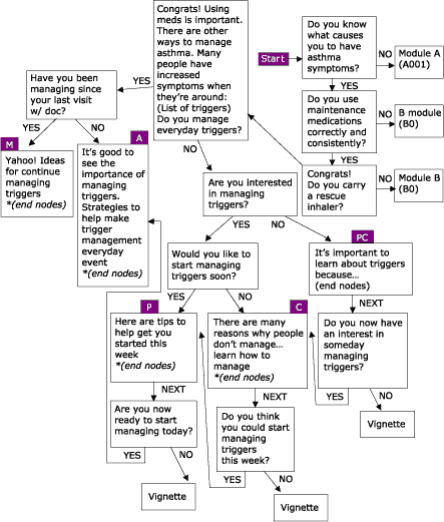
Managing Environmental Triggers, one of four modules within the Asthma Management Demonstration Project. PC = Precontemplation; C = Contemplation; P = Preparation; A = Action; and M = Maintenance.

**Figure 5:**
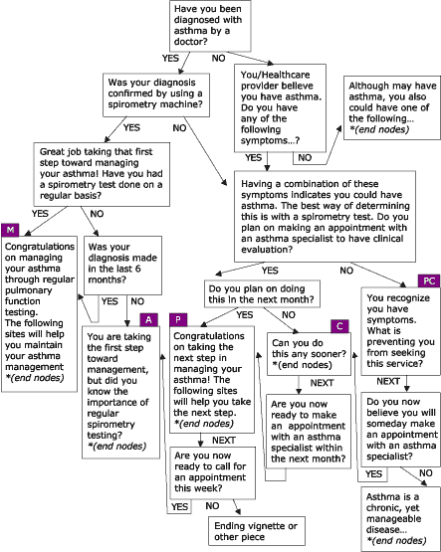
Accessing Asthma Services, one of four modules within the Asthma Management Demonstration Project. PC = Precontemplation; C = Contemplation; P = Preparation; A = Action; and M = Maintenance.

**Figure 6:**
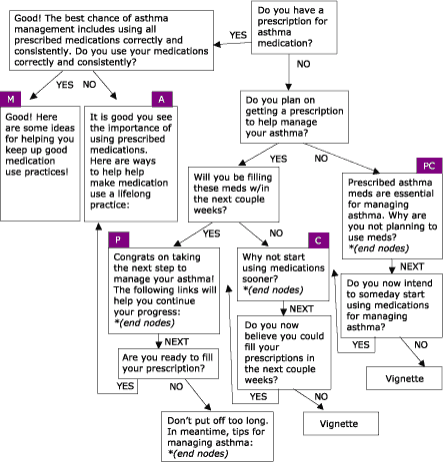
Using Prescription Drugs Properly, one of four modules within the Asthma Management Demonstration Project. PC = Precontemplation; C = Contemplation; P = Preparation; A = Action; and M = Maintenance.

**Figure 7:**
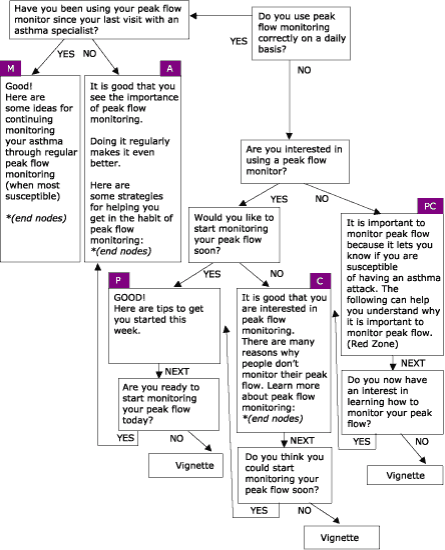
Regular Peak Flow Monitoring, one of four modules within the Asthma Management Demonstration Project. PC = Precontemplation; C = Contemplation; P = Preparation; A = Action; and M = Maintenance.

Many people living with asthma perceive asthma management to be limited to symptom control. This perception is antithetical to the goals of asthma management established by health educators and medical professionals. In response, the Asthma Management Demonstration Project was developed by the Office of Health Promotion and Health Education at Western Michigan University, with the purpose of managing behaviors and conditions that lead to asthma-related consequences among employees and students of Western Michigan University. Four modules based on the eHealth Behavior Management model were created to address the issues of asthma management: peak flow monitoring, accessing asthma services, using prescription asthma medications properly, and managing environmental factors that trigger asthma attacks. Like the WIC nutrition education project, four algorithms were created using empowering statements, persuasive arguments, and transactional questioning to stage users based on stage of readiness to change behavior. The modules are available online from URL: http://www.wmich.edu/shc/HPAE/awp/homeset.html*. The underlying algorithms used for this project are depicted below. Figure 4 shows the module on managing environmental triggers; Figure 5 shows the module on accessing asthma services; Figure 6 shows the module on using prescription drugs properly; and Figure 7 shows the module on monitoring peak flow regularly. The asthma project differs from the nutrition education project in the way that algorithms link with one another. For example, in the module "Managing Environmental Triggers," initial questions determine if users are actually ready to start managing triggers or if users need to focus first on accessing medical care and using prescription asthma medications properly, both of which are separate but linked modules within the project (Figure 4).

Two kiosks (stand-alone booths) placed in strategic locations on the university campus serve as the mode for delivering the asthma wellness education modules. Kiosks were chosen and designed to maximize ease of use, ensure information usefulness, and enhance user satisfaction with the educational experience. SiteKiosk database software (Provisio, Muenster, Germany) was used to collect data based on user responses and to send a secure e-mail transmission of the kiosk's daily activities. Log files are downloaded from e-mails and then uploaded to a database for analysis, which allows for a deeper level of analysis of interactions between users and kiosks. 

The Table presents preliminary data from 133 days of kiosk operation. Based on analysis of log files, it was determined that at least 20 navigations within the Web site are required to represent a quality use, which includes an initiating navigation, navigation within the module pages, and navigation into at least one end node within a module. During the 133 days analyzed, there were 220 total quality uses with an average of 1.66 quality uses per day with 1.72 end-node visits per quality use. The module "Accessing Asthma Services" was the most popular module accessed, likely due to a variety of reasons including the broad nature of the topic and the location of the menu item (at the top of the module selection page). Analysis of the actual path each user completes as well as progress in intent toward stages of change will be the purpose of final evaluation and analysis.

### Preventing HIV through social-wellness building in South Africa

It is estimated 25 to 28 million of the 34 to 46 million individuals living with human immunodeficiency virus/acquired immunodeficiency syndrome (HIV/AIDS) live in sub-Saharan Africa (33). It is further estimated that five million people living in South Africa are infected with HIV, including 20% of individuals aged 15 to 49 years, representing one of the largest HIV-infected populations in the world (34). The transmission of HIV/AIDS in South Africa is linked primarily to sexual contact between opposite sexes. Women are at greater risk of HIV because of physical and sexual violence, unequal distribution of power among relationships between men and women resulting from male-dominated cultures, migratory labor practices of male heads of households, lack of access to relevant and accurate information about HIV/AIDS, and economic dependence of women due to lack of economic power (35-38).

**Figure 8:**
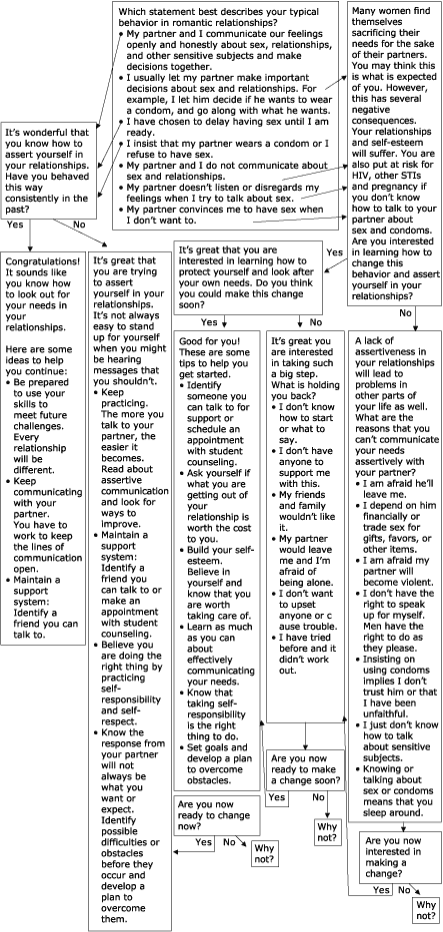
HIV/AIDS prevention assertive communication module currently being developed for South African women at the University of Port Elizabeth, South Africa.

The eHealth Behavior Management Model is the basis for an HIV/AIDS-prevention assertive-communication module currently being developed for South African women at the University of Port Elizabeth, South Africa. Central to the project is the goal of helping women to move toward more active behaviors associated with open and assertive communication about sexual relationships and practices. The empowering statements, persuasive arguments, and transactional questions are now being pilot-tested for validity, usability, and cultural translation with subsets of women at the University of Port Elizabeth (Figure 8). Web design and applicability issues are also being addressed.

Many challenges exist in applying this model to a population outside the United States, including different Internet practices and accessibility issues and the lack of available and culturally appropriate end nodes. Access to the Internet differs dramatically from that in the United States, even though South Africa has the highest availability of Internet access of any country in Africa. Internet connection is especially lacking in rural South Africa; high levels of poverty prevent South Africans from building the infrastructure necessary for Internet connections; individuals from populations at higher risk are often not computer-literate, and costs associated with per-minute connection to the Internet proportionately far exceed costs found in the United States. The major end-node barrier is the lack of existing Web pages specific to South African cultures and languages. Most existing Web pages are based in the United States, which limits the number of appropriate end nodes. This project requires the development and cultural adaptation of each end node, which in itself is a time-consuming process. Initial focus group results have indicated high interest in and acceptance of this Web-based assertive-communications program as a viable method for behavior change among the targeted population.

## Conclusion

The potential use of the eHealth Behavior Management Model is both positive and broad, especially considering that the Internet continues to be a viable avenue for health-related resources. Expanded use of this model across other chronic disease management issues, such as diabetes control, and across other primary prevention issues, such as daily folic acid intake or weight management, needs to occur to truly validate the extent to which this model is applicable.

The benefits so far observed from implementation of the eHealth Behavior Management Model are numerous. The behavior management and behavior change aspects of this model are inherently evident. Early results have found the model attracts people who are in the more active stages of readiness to change (i.e., Preparation, Action, and Maintenance). This may be because users who seek information from the Internet are more likely to change or to be getting ready to change behaviors. This does not discount the use of the model as a method within a comprehensive behavior management or behavior change approach, but rather suggests it may be an effective means for providing change-related skills to a subset within a population at risk. Equally important is the ability to use the model as a diagnostic tool for identifying the stages that users are in and the issues in which they are most interested. Being Web-based allows for data collection and analysis of path movement, including specific end nodes visited. Analyses of user traffic may provide program planners with a greater understanding of the key issues that seem to be of interest or have the greatest impact on preventing users from progressing toward an active stage of change.

Internet-based approaches allow for reaching large numbers of people at relatively low costs. The rapid increase in Internet use over the past decade attests to the expanding interest in using the Internet as an avenue for conveying health-related messages and skills to consumers. The eHealth Behavior Management Model is built on providing better and more appropriate access to this information. As with all approaches, though, challenges do exist. The model directs users to existing end nodes, and much time is required to maintain and validate end-node links. Because end nodes are maintained outside the control of the eHealth Behavior Management Model*,* there is a greater likelihood that these external sources information may not be available in the event of end-node host-server errors. Much of this uncertainty can be avoided through reciprocal communication with the end-node site owners, contingency plans for replacing end nodes in the event of failure, and weekly or daily monitoring of end-node functioning.

Use of predetermined end nodes deviates from more traditional tailored communication approaches, decreasing the ability to direct users to tailored messages based on user-interactive input. The trade-off is the benefit of the breadth of potential existing end nodes and the lower costs compared to those of developing computer-based expert systems. When used in conjunction with other forms of behavior change management, such as follow-up health counseling, the eHealth Behavior Management Model can play an important role in initiating client movement along the stages-of-change continuum.

The usefulness and impact of this model are currently under investigation, with initial results showing the eHealth Behavior Management Model to be both useful and effective with intent toward health behavior change. It is recommended this model be adopted to and evaluated with other health behavior management and change issues.

## Figures and Tables

**Table:** Preliminary Asthma Management Demonstration Project Usage Statistics, Sept 26, 2003–Apr 5, 2004, Kalamazoo, Mich

Total navigations	4404
Average navigations per day	33.1
Days of data collection	133
Module A-related navigations	868
Module B-related navigations	575
Module C-related navigations	306
Module D-related navigations	453
End nodes accessed	379
Estimated quality uses	220.2
End nodes accessed per quality use	1.7
Estimated quality uses per day	1.7

## References

[1] Lalonde M (1974). A new perspective on the health of Canadians: a working document.

[2] Mokdad AH, Marks JS, Stroup DF, Gerberding JL (2004). Actual causes of death in the United States, 2000. JAMA.

[3] Prochaska JO, DiClemente CC (1983). Stages and processes of self-change of smoking: toward an integrative model of change. J Consult Clin Psychol.

[4] Bensley RJ, Bensley RJ, Brookins-Fisher J (2003). Community health education methods: a practical guide.

[5] (2004). Metamend. How big is the Internet? How fast is the Internet growing [Internet]?.

[6] (2002). Nua Internet Surveys. How many online [Internet]?.

[7] (2004). Pew Internet & American Life Project. Older Americans and the Internet [Internet].

[8] Bensley RJ, Underhile R, de Jager AC, Bettin K, Mercer N, Brusk JS Application of the eHealth Behavior Change and Management Model.

[9] Billing AS, Atkinson NL, Gold R Assessing the needs of food stamp recipients in five Maryland counties.

[10] U.S. Department of Health and Human Services (2000). Healthy people 2010: understanding and improving health.

[11] (2003). Pew Internet & American Life Project. America’s online pursuits: the changing picture of who’s online and what they do [Internet].

[12] Taylor H (2003). Those with Internet access to continue to grow but at a slower rate [Internet].

[13] Taylor H (2002). Internet penetration at 66% of adults (137 million) nationwide [Internet].

[14] Bock B, Graham A, Sciamanna C, Krishnamoorthy J, Whiteley J, Carmona-Barros R (2004). Smoking cessation treatment on the Internet: content, quality, and usability. Nicotine Tob Res.

[15] Bensley RJ, Lewis J (2002). Analysis of Internet-based health assessments. Health Promot Pract.

[16] Kreuter M, Farrell D, Olevitch L, Brennan L (1999). Tailoring health messages: customizing communication with computer technology.

[17] Kreuter MW, Jacobsen HA, McDonald EM, Carlson-Gielen A, Bensley RJ, Brookins-Fisher J (2003). Developing computerized tailored health messages. Community health education methods: a practical guide.

[18] Kreuter M, Vehige E, McGuire AG (1996). Using computer-tailored calendars to promote childhood immunization. Public Health Rep.

[19] Kreuter M, Bull F, Clark E, Oswald D (1999). Understanding how people process health information: a comparison of tailored and untailored weight loss materials. Health Psychol.

[20] Rimer BK, Orleans CT, Fleisher L, Cristinzio S, Resch N, Telepchak J (1994). Does tailoring matter? The impact of a tailored guide on ratings and short-term smoking-related outcomes for older smokers. Health Educ Q.

[21] Rimer BK, Orleans CT (1994). Tailoring smoking cessation for older adults. Cancer.

[22] Strecher V, Wang C, Derry H, Wildenhaus K, Johnson C (2002). Tailored interventions for multiple risk behaviors. Health Educ Res.

[23] Velicer WF, Prochaska JO, Fava JL, Norman GJ, Redding CA (1998). Smoking cessation and stress management: applications of the Transtheoretical Model of behavior change. Homeostasis.

[24] Ajzen I (2002). Theory of planned behavior [Internet].

[25] Cassell MM, Jackson C, Cheuvront B (1998). Health communication on the Internet: an effective channel for health behavior change?. J Health Commun.

[26] Reed GR, Velicer WF, Prochaska JO (1997). What makes a good staging algorithm: examples from regular exercise. Am J Health Promot.

[27] DiClemente CC, Prochaska JO, Fairhurst S, Velicer WF, Rossi JS, Velasquez M (1991). The process of smoking cessation: an analysis of precontemplation, contemplation and contemplation/action. J Consult Clin Psychol.

[28] Velicer WF, Fava JL, Prochaska JO, Abrams DB, Emmons KM, Pierce J (1995). Distribution of smokers by stage in three representative samples. Prev Med.

[29] Vallis M, Ruggiero L, Greene G, Jones H, Zinman B, Rossi S (2003). Stages of change for healthy eating in diabetes: relation to demographic, eating-related, health care utilization, and psychosocial factors. Diabetes Care.

[30] Brusk JS, Bensley RJ (2004). Midyear evaluation report 2003-2004 [Internet].

[31] Bensley RJ Nutrition education on the Internet.

[32] (2002). Centers for Disease Control and Prevention. Behavioral Risk Factor Surveillance System Survey Data.

[33] (2003). World Health Organization. AIDS epidemic update: December 2003 [Internet].

[34] (2004). USAID. Country Profile: HIV/AIDS South Africa [Internet].

[35] Dunkle KL, Jewkes RK, Brown HC, Gray GE, McIntryre JA, Harlow SD (2004). Gender-based violence, relationship power, and risk of HIV infection in women attending antenatal clinics in South Africa. Lancet.

[36] Gilbert L, Walker L (2002). Treading the path of least resistance: HIV/AIDS and social inequalities — a South African case study. Soc Sci Med.

[37] Health24. Women’s vulnerability to HIV infection [Internet].

[38] Lurie MN, Williams BG, Zuma K, Mkaya-Mwamburi D, Garnett G, Sturm AW (2003). The impact of migration on HIV-1 transmission in South Africa: a study of migrant and nonmigrant men and their partners. Sex Transm Dis.

